# Standardization of Synthetic Biology Tools and Assembly Methods for *Saccharomyces cerevisiae* and Emerging Yeast Species

**DOI:** 10.1021/acssynbio.1c00442

**Published:** 2022-08-08

**Authors:** Koray Malcı, Emma Watts, Tania Michelle Roberts, Jamie Yam Auxillos, Behnaz Nowrouzi, Heloísa Oss Boll, Cibele Zolnier Sousa do Nascimento, Andreas Andreou, Peter Vegh, Sophie Donovan, Rennos Fragkoudis, Sven Panke, Edward Wallace, Alistair Elfick, Leonardo Rios-Solis

**Affiliations:** † Institute for Bioengineering, School of Engineering, University of Edinburgh, Kings Buildings, EH9 3BF Edinburgh, United Kingdom; ‡ Centre for Synthetic and Systems Biology (SynthSys), University of Edinburgh, Kings Buildings, EH9 3BD Edinburgh, United Kingdom; § School of Biological Sciences, University of Edinburgh, Kings Buildings, EH9 3JW Edinburgh, United Kingdom; ∥ Department of Biosystems Science and Engineering, ETH Zürich, 4058 Basel, Switzerland; ⊥ Institute of Cell Biology, School of Biological Sciences, University of Edinburgh, Kings Buildings, EH9 3FF Edinburgh, United Kingdom; # Department of Genetics and Morphology, Institute of Biological Sciences, University of Brasília, Brasília, Federal District 70910-900, Brazil; × Department of Biotechnology, Lorena School of Engineering, University of São Paulo, Lorena, São Paulo 12602-810, Brazil; + Edinburgh Genome Foundry, University of Edinburgh, Kings Buildings, Edinburgh EH9 3BF, United Kingdom; ⊗ School of Natural and Environmental Sciences, Newcastle University, Newcastle upon Tyne NE1 7RU, United Kingdom

**Keywords:** standardization, characterization, biological parts, yeast toolkits, synthetic biology, automation

## Abstract

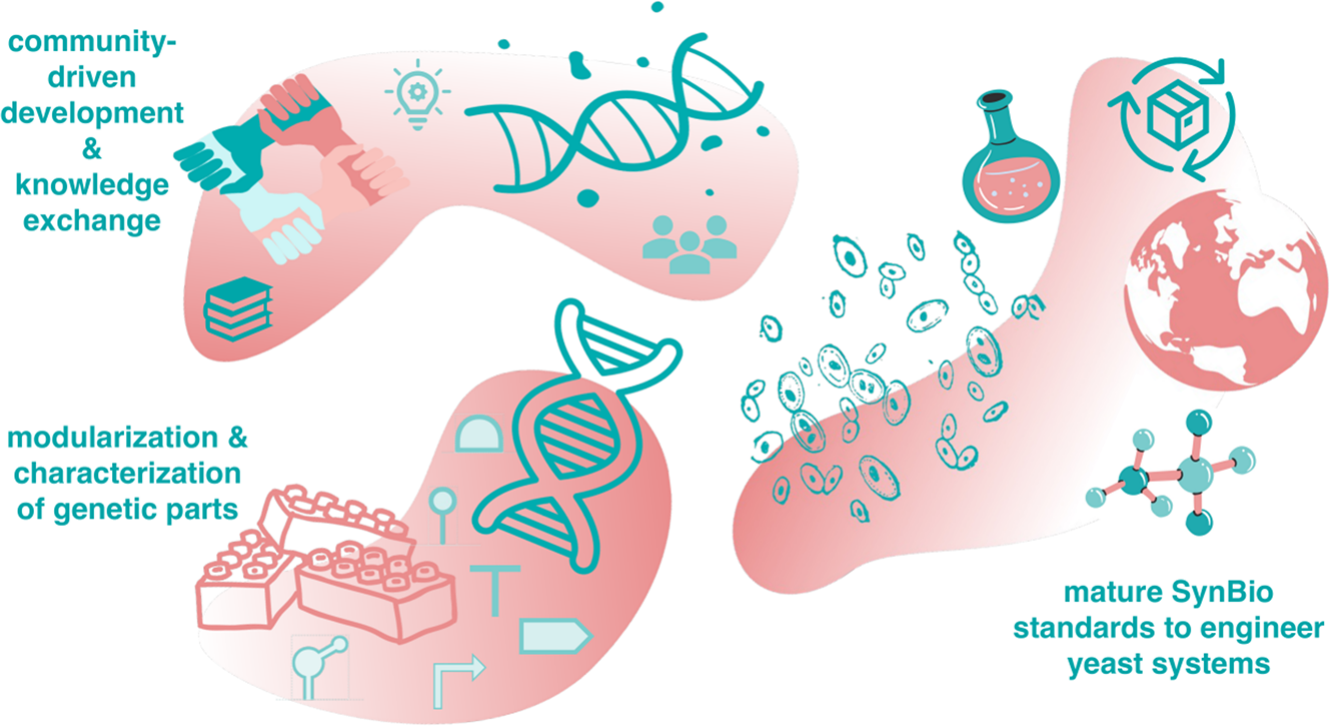

As redesigning organisms using engineering principles is one of the purposes of synthetic biology (SynBio), the standardization of experimental methods and DNA parts is becoming increasingly a necessity. The synthetic biology community focusing on the engineering of *Saccharomyces cerevisiae* has been in the foreground in this area, conceiving several well-characterized SynBio toolkits widely adopted by the community. In this review, the molecular methods and toolkits developed for *S. cerevisiae* are discussed in terms of their contributions to the required standardization efforts. In addition, the toolkits designed for emerging nonconventional yeast species including *Yarrowia lipolytica*, *Komagataella phaffii*, and *Kluyveromyces marxianus* are also reviewed. Without a doubt, the characterized DNA parts combined with the standardized assembly strategies highlighted in these toolkits have greatly contributed to the rapid development of many metabolic engineering and diagnostics applications among others. Despite the growing capacity in deploying synthetic biology for common yeast genome engineering works, the yeast community has a long journey to go to exploit it in more sophisticated and delicate applications like bioautomation.

## 1 Introduction

Each widely used technology, from architecture to information technology to synthetic biology, has its standards that have evolved. Standards are the common language to increase compatibility, interoperability, and the quality of the related technology. Specifications of the standards are built up by a consensus view among the communities and/or institutions, and these specifications are the main outcome of the standardization process.^1,2^ In multidisciplinary fields such as engineering, standards have been a cornerstone to reach a global, coordinated way for obtaining impactful outputs.^3,4^ Years ago, Endy (2005) proposed the three pillars of SynBio to the community in correlation to engineering: standardization, decoupling, and abstraction.^5^ However, despite the efforts, key developments in standardizing biological constructs and methodologies are yet to be achieved in SynBio.^6^ Uniting behind a consolidated set of SynBio standards will likely serve to accelerate translation to impact commercial applications. Part of this entails the creation of toolkits, consisting of a well-characterized library of standardized DNA minimal parts, such as promoters and terminators of different strengths and coding sequences/tags of different functions as well as standardized assembly methods to form more complicated genetic circuits.

Not surprisingly, early SynBio studies were carried out in *Escherichia coli*, as a versatile microbial chassis.^7−10^ Hence, many standardized languages, concepts, genetic parts, and molecular tools have been initially developed for this organism.^11,12^ However, there are a set of functionalities that are simply inaccessible in such systems, such as epigenetic control and post-translational protein modifications, that necessitate the use of a more appropriate chassis like yeast and their respective toolkits.^13,14^

The fascinating biochemical and genetic features of *Saccharomyces cerevisiae* have made it a popular eukaryotic model organism for synthesizing a wide range of biological, biomaterial, and chemical products.^15−19^ While *S. cerevisiae*’s biodesign studies mainly focus on top-down strategies, a notable bottom-up approach is the total biodesign and resynthesis of the yeast genome in the Sc 2.0 project.^20−22^ Part of this success is due to strong efforts by the *S. cerevisiae* community to drive the development and adoption of several DNA toolkits that have become common to yeast SynBio research.

**Figure 1:**
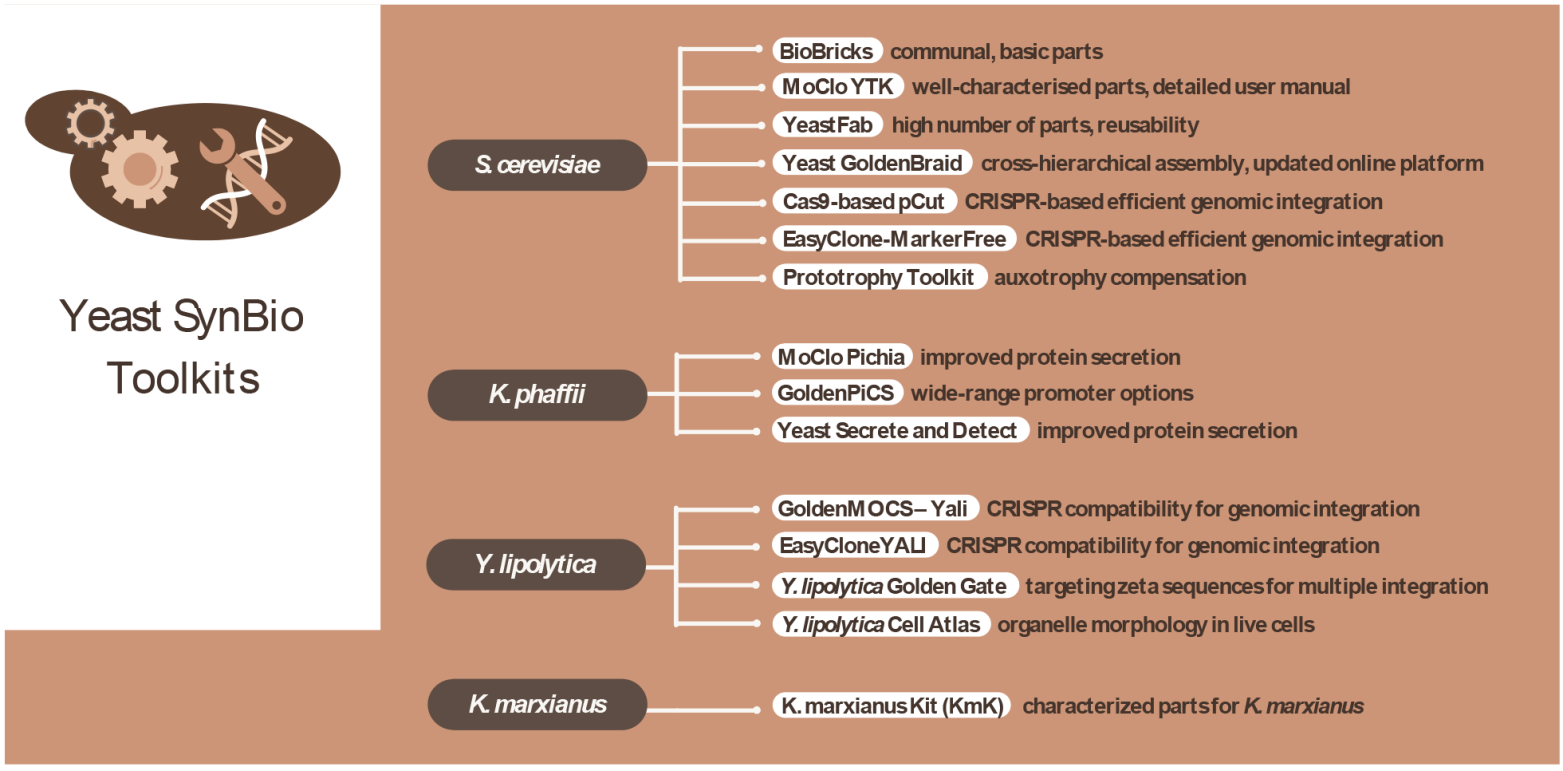
Overview of the selected yeast synthetic biology toolkits mapped in this review.

The scope of this review is to explore the best SynBio practices through a more detailed investigation of the most used *S. cerevisiae* toolkits if not describing all the evolved tools (Figure 1). The deployment of these toolkits for nonconventional yeasts, like *Kluyveromyces marxianus*, *Yarrowia lipolytica*, and *Komagataella phaffi* (*Pichia pastoris*) will be discussed. By describing and sharing the successful impact of these toolkits, best practice lessons should be reflected onto other microbial chassis and the further advancement of SynBio research and innovation.

## 2 Selected *S. cerevisiae* Synthetic Biology Toolkits

### 2.1 *S. cerevisiae* BioBricks

BioBricks are standardized and interchangeable parts representing functional biological units such as promoters, ribosomal binding sites (RBS), terminators, protein domains, tags, or protein-coding sequences among others.^23,24^ BioBrick parts are collected in the Registry of Standard Biological Parts, one of the largest repositories of parts in synthetic biology. This online catalog^25^ envisioned to organize and document parts encoding biological functions works as the central resource of DNA encoding parts to all participants in the International Genetically Engineered Machine (iGEM) competition.^26^

The requirements associated with defining a “standard” biological part include assembling parts together as well as the associated sequence specifications. In this regard, the BioBricks parts can be assembled to construct more complex DNA structures like expression vectors to provide flexible modularity. Here, the type II restriction enzymes are used to create compatible ends between adjacent parts. Therefore, the parts have prefixes and suffixes, being EcoRI and Xbal as prefixes and SpeI and PstI as suffixes. Sequential joining reaction can then be used for the idempotent assembly of parts.^27^

Employing BioBricks is relatively simple, and there are many well-written and detailed protocols available for the users on the registry Web site.^25^ However, in any assembly step, only two parts can be joined to ensure that the final product is in the correct order. Furthermore, the 6 bp enzyme restriction recognition sites must not be present elsewhere in the sequence. Hence, these forbidden sites are removed in a “domestication” step. While the sequences are confirmed by iGEM, the quality of the parts in the registry is variable. This is because of the presence of a diverse pool of submissions which have not been necessarily curated or lack sufficient characterization data. iGEM headquarters work on a quality control (QC) check for the parts listed and release the QC information containing the results of sequencing, gel electrophoresis, growth plate, and antibiotic test plate.^28,29^ Nevertheless, the sheer number of parts received makes it impractical for iGEM staff to have a direct role in parts characterization and functional validation.^26^ Pragmatically, the actual impact of part quality variability is moot as low-quality parts are rejected by the user community.

**Figure 2:**
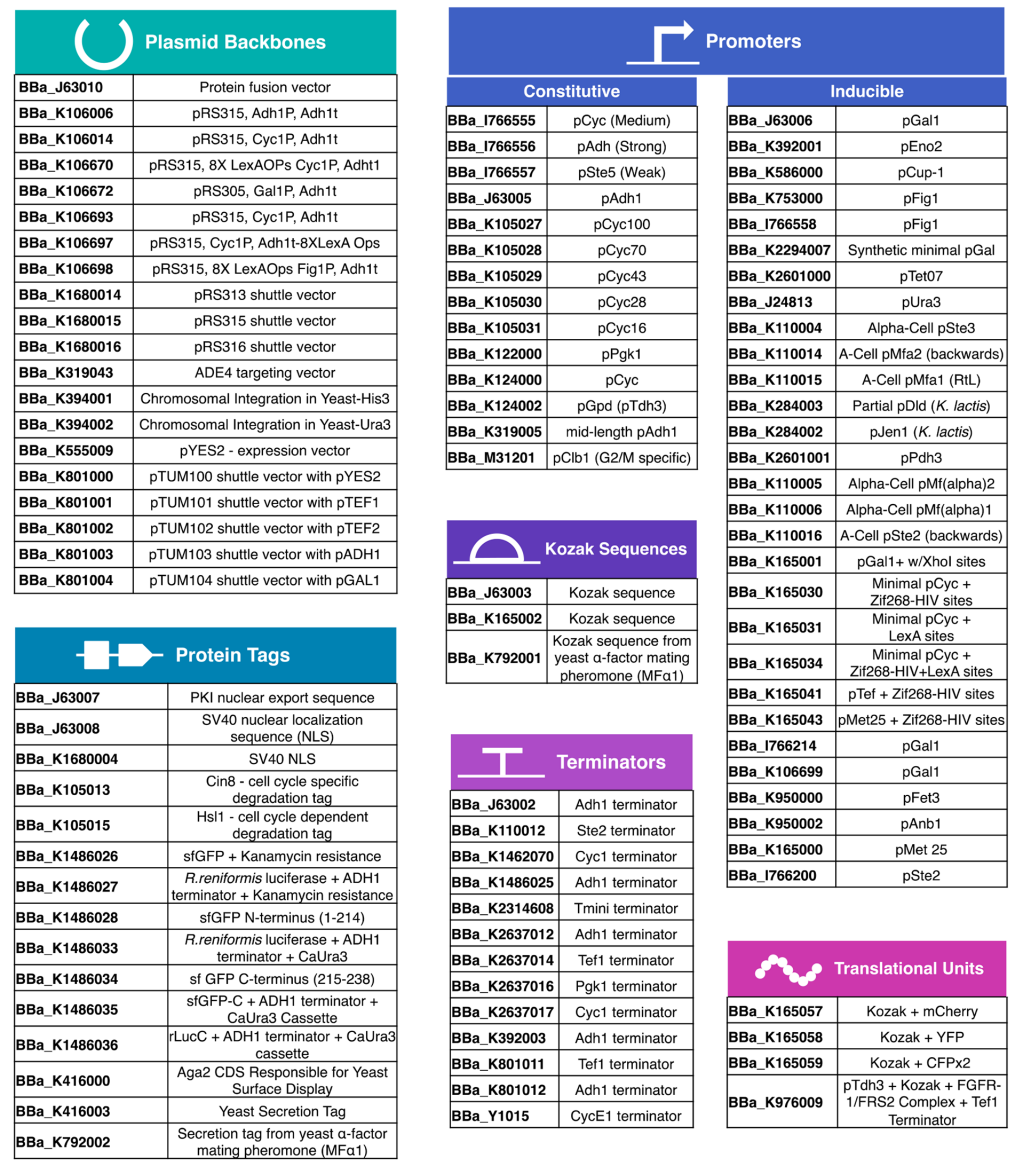
Standard biological parts listed for *S. cerevisiae* at the iGEM Registry.^30^

While the database is focused mainly on bacteria (*i.e.*, *E. coli*), there is a relatively small collection of characterized *S. cerevisiae* parts available as shown in Figure 2. This makes up the *S. cerevisiae* kit^30^ rather small compared to the over 20 000 parts documented in the iGEM Registry.

Yeast BioBricks Assembly (YBA) is one of the early examples of standardization of yeast expression vector assembly using a single restriction enzyme and BioBrick parts.^31^ Later, Stocivek *et al.* (2015) developed EasyClone 2.0 vectors which is a new set of genome-integrating EasyClone vectors^32^ using standardized BioBrick parts.^33^ While EasyClone vectors contain auxotrophic markers,^32^ EasyClone 2.0 vectors contain either auxotrophic markers or dominant selective markers, relying on drop-out media, antibiotics, or the ability to grow on alternate nitrogen sources for selection of edited strains.^33^ To remove the markers from the genome, the Cre/LoxP marker recycling system can then be used.^34^ However, later on, the same group^35^ reported a marker-free vector suite called EasyClone-MarkerFree which we will discuss in detail in this review. Though EasyClone vectors were designed to target 11 well-defined genomic regions, they were still not suitable for prototrophic strains. Therefore, six different dominant selection markers like nourseothricin, hygromycin were added to EasyClone 2.0 vectors to make them suitable for prototrophic industrial yeasts.

Parallel to iGEM Registry, the Joint BioEnergy Institute has a repository of information about biological parts, plasmids, and strains, known as the Inventory of Composable Elements (JBEI-ICEs).^36^ This open-source, community-driven platform^37^ currently hosts more than 300 yeast-related plasmids submitted by users. Although relatively fewer parts are available in this repository, it is quite well-organized with detailed information, including graphical annotations and creator’s contact details, available for every single part.

### 2.2 Modular Cloning Systems (MoClo)

**Figure 3:**
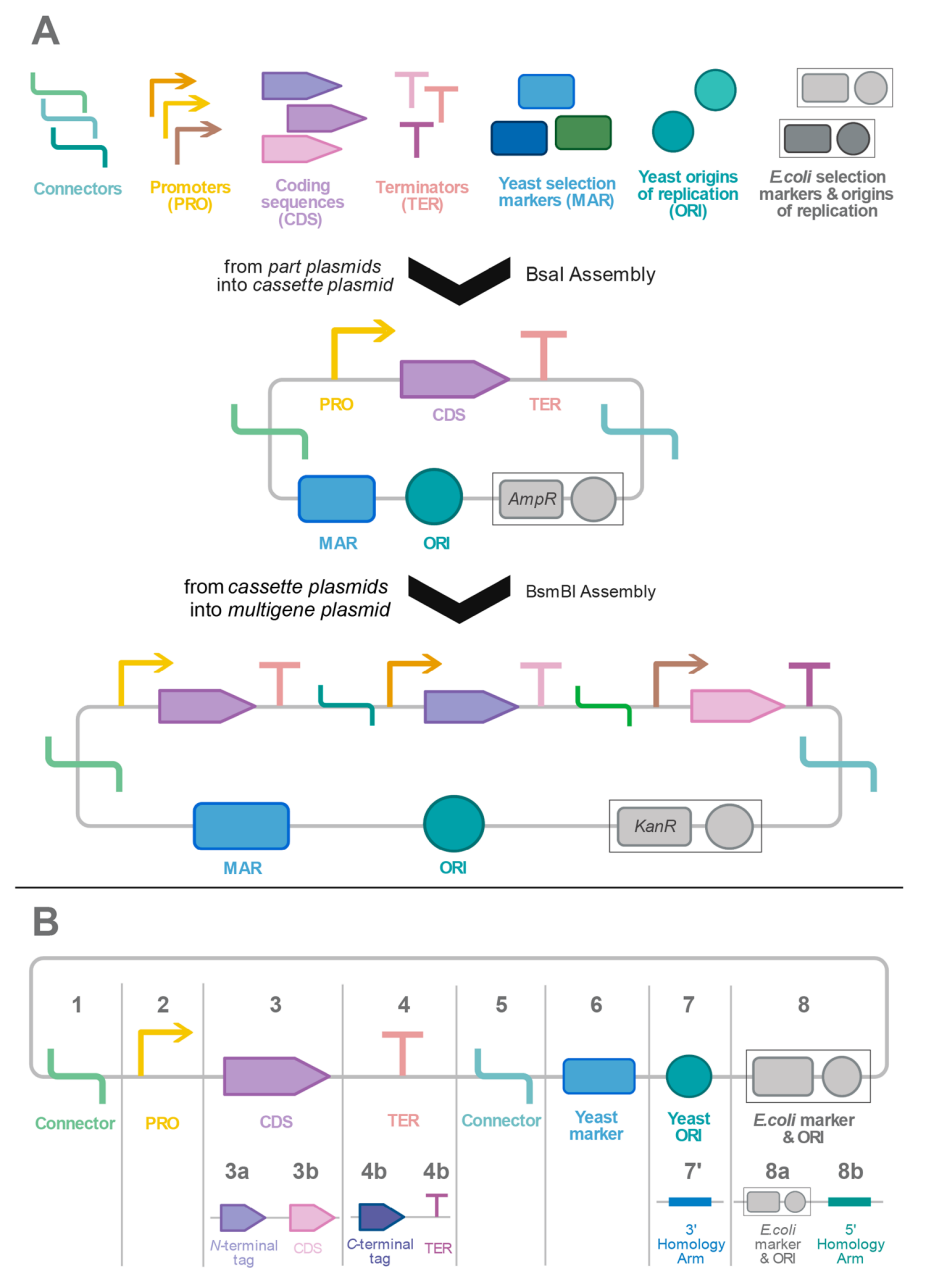
Hierarchical assembly strategy in MoClo YTK. (A) Golden Gate-based assembly mechanism of the toolkit. The parts are first generated *via* PCR or synthetic DNAs are used as sources and they are kept in the part plasmids. In the next level, the parts are assembled by using the BsaI type IIS restriction enzyme to create transcriptional units (TU) that usually contain a promoter, coding sequence (CDS), and terminator. At this level, plasmids have an ampicillin-resistance marker (*AmpR*). When needed, multiple TUs can be assembled by using the BsmBI type IIS restriction enzyme to obtain a multigene plasmid. At this level, plasmids have a kanamycin-resistance marker (*KanR*). (B) The part types used in MoClo YTK. Each number represents a particular type. The types can be further modularized. Type 3 can be split into two so that an N-terminal tag (Type 3a) can be used with the CDS (Type 3b). Likewise, Type 4 can be either a C-terminal tag (Type 4a) or terminator (Type 4b) for genomic integration, and Type 7, which is used for yeast origin of replication (ORI), can be replaced with a 3′ homology arm, where Type 8b can be used as a 5′ homology arm. Then, the construct is linearized with homology arms at each end.

Developed in 2011 by Weber *et al.*, MoClo enabled a hierarchical assembly of multiple genes in eukaryotes.^38^ The system is based on the Golden Gate assembly method^39^ and uses type IIS restriction enzymes to create unique 4-base overhangs for multipart assembly reactions.^38^ Being a modular cloning system, at build level 0, parts like promoters, 5′ untranslated regions, signal peptides, coding sequences, and terminators are selected from the parts library. At level 1, these parts are combined into transcriptional units, and at level 2, these transcriptional units are assembled into multigene constructs. To facilitate assembly, each part has a unique upstream and downstream overhang pair, and a complete cassette can be assembled in order. In 2015, Lee *et al.* adopted the MoClo approach for *S. cerevisiae* and developed a highly characterized and easy-to-use toolkit, MoClo Yeast Toolkit (YTK) (Figure 3A) that has been widely used.^40−43^

This toolkit consists of 96 standardized parts, including promoters, terminators, peptide tags, origins of replication, and genome-editing tools, all available in a single 96-well plate format from Addgene.^44^ The available parts, such as promoters were well-characterized, included a range of relative strengths and were easily interchangeable. Eight types of parts were identified with numbers and detailed documentation for the use of each type was given.^43^ The parts could be further modularized for specific applications. For instance, as shown in Figure 3B, Type 3 normally represents a coding sequence (CDS) but could be split into two, containing the N-terminal tag and CDS. The assembly could also be designed for genomic integration by adding 3′ and 5′ homology arms (Figure 3B).

Although both MoClo and standard BioBricks assembly methods rely on the robust and well-understood Golden Gate,^45−47^ MoClo was developed in such a way that up to six pieces of DNA could be efficiently assembled in a single step. MoClo assembly process is relatively fast, taking around 3 days to construct a multigene plasmid. Another advantage of the MoClo YTK is the availability of the parts in plasmids in a reusable and easy to share 96-well format. In addition, the use of assembly connector sequences has allowed MoClo YTK parts to be compatible with parts from other toolkits. Assembly connectors harbor the enzyme recognition sites but also contain homology sequences for recombination-based and isothermal assembly based toolkits. The restriction enzymes BsaI and BpiI/BbsI cut a short distance from the recognition sequence and hence, making them compatible with other Golden Gate-based methods, like BioBricks, as ends can be user-defined. Apart from that, the MoClo assembly can be readily automatable,^48,49^ more easily achievable through recent advances in automation and the facilities of biofoundries.^50^ Besides, MoClo-compatible software is offered by the biofoundries to users to facilitate the automation process. For instance, Edinburgh Genome Foundry offers a suite of free SynBio software^51^ including the Collection of Useful Biological Apps repository (CUBA; https://cuba.genomefoundry.org/) and Design-And-Build (DAB; https://dab.genomefoundry.org/) for MoClo-compatible DNA parts assembly and quality control prior to automation. This is also discussed below with more detail.

A key advantage of the MoClo YTK was that several of the DNA parts (*i.e.*, promoters, terminators) and their effect on expression were characterized using a single, well-standardized methodology allowing the users to easily compare between them. To characterize the promoters, relative strength was measured with two different fluorescent marker proteins, mRuby2^52^ and Venus,^53^ by normalizing the raw fluorescence values to the OD600 values of the cultures.^43^ Terminators were characterized with three different markers, mTurquoise2,^54^ Venus, and mRuby2, each in combination with three different promoters. The difference in expression levels between promoter-terminator pairs offered an understanding of how to best utilize these parts. Cassettes were also made with fluorescent markers for protein degradation tags Ubi-M (weak), Ubi-Y (medium), and Ubi-R (strong).^55^ The plasmid copy number based on the origin of replication (CEN/ARS, low copy; 2-μ, high copy) were also evaluated for how they affected the expression level. Similar to promoter characterization, mRuby2 and Venus reporter proteins were used for copy number characterization with normalized fluorescence values for cell size after measuring the cell cultures at exponential phase on a flow cytometer.^43^ Furthermore, the researchers investigated intrinsic cell–cell variability.^56^ The effect of plasmid copy number was also demonstrated by integrating single or multiple plasmids and integrating at single or multiple loci, whereby using a higher copy number resulted in larger variability in the results.^43^ Thoroughly characterized transcriptional libraries facilitate optimization of expression as there is information available about how parts interact in a construct as well as just information about the part itself.

MoClo YTK parts are unusually well-characterized, unlike iGEM parts, the information about the part functions is given. However, there are limits. The parts were characterized in only synthetic defined media; therefore, the effects of different media types on gene expressions driven by different promoter-terminator pairs are not known. Also, a limited number of *S. cerevisiae* strains, BY4741 and 4742, were used for the part characterization. Therefore, diverse outcomes might be obtained from other widely used yeast strains, such as CEN.PK, SK1, W303, Ethanol Red, and EC1118.^57^ For this reason, individual promoter-terminator pairs should be tested for sensitive studies or for different conditions as suggested by the authors.^43^ Fortunately, more reports about the use of MoClo on different yeast strains have been published. For instance, using MoClo YTK components, CEN.PK133-derived yeast strains were designed for *de novo* nepetalactone synthesis.^58^ Besides, CEN. PK2-1C strain was used to produce the artificial deazaflavin cofactor FOP in yeast engineered with the help of MoClo YTK.^59^ Although an absolute comparison cannot be made, the relative performance of the toolkit and the parts can be estimated considering these and similar studies making use of MoClo YTK in different conditions and strains.

Yet, the application of MoClo YTK has been extended to the development of other SynBio toolkits for yeast. For instance, a light-inducible gene expression regulation system, called yeast optogenetic toolkit (yOTK), was developed as an expansion of MoClo YTK.^60^ To this end, two artificial transcription factors, ZDBD-CRY2 and VP16-CIB1, were created as CDS (Type 3 part) in MoClo YTK and a corresponding promoter pZF was used as a promoter (Type 2 part) in the toolkit.^60^ MoClo YTK was also used to build a GPCR-sensor Toolkit,^61^ providing 42 new parts, such as promoters, upstream activating sequences, repressors, GPCR subunits, transcription factors, and reporter proteins for tunable GPCR signaling pathways for yeast.^62^ Recently, MoClo YTK has been expanded for CRISPR-based application by introducing 35 new plasmids designed for this purpose.^63^ Using this CRISPR-based MoClo YTK, authors also constructed a Csy4-multiplexed gRNA array to be used for simultaneous genome editing studies. Moreover, a design tool in the form of an R-shiny app was developed to mitigate the hassle of designing new MoClo parts.^63^ Although the 35 plasmids developed in this study have been deposited into Addgene,^64^ the toolkit is not available in a well-plate format; therefore, each plasmid should be obtained separately.

### 2.3 YeastFab

**Figure 4:**
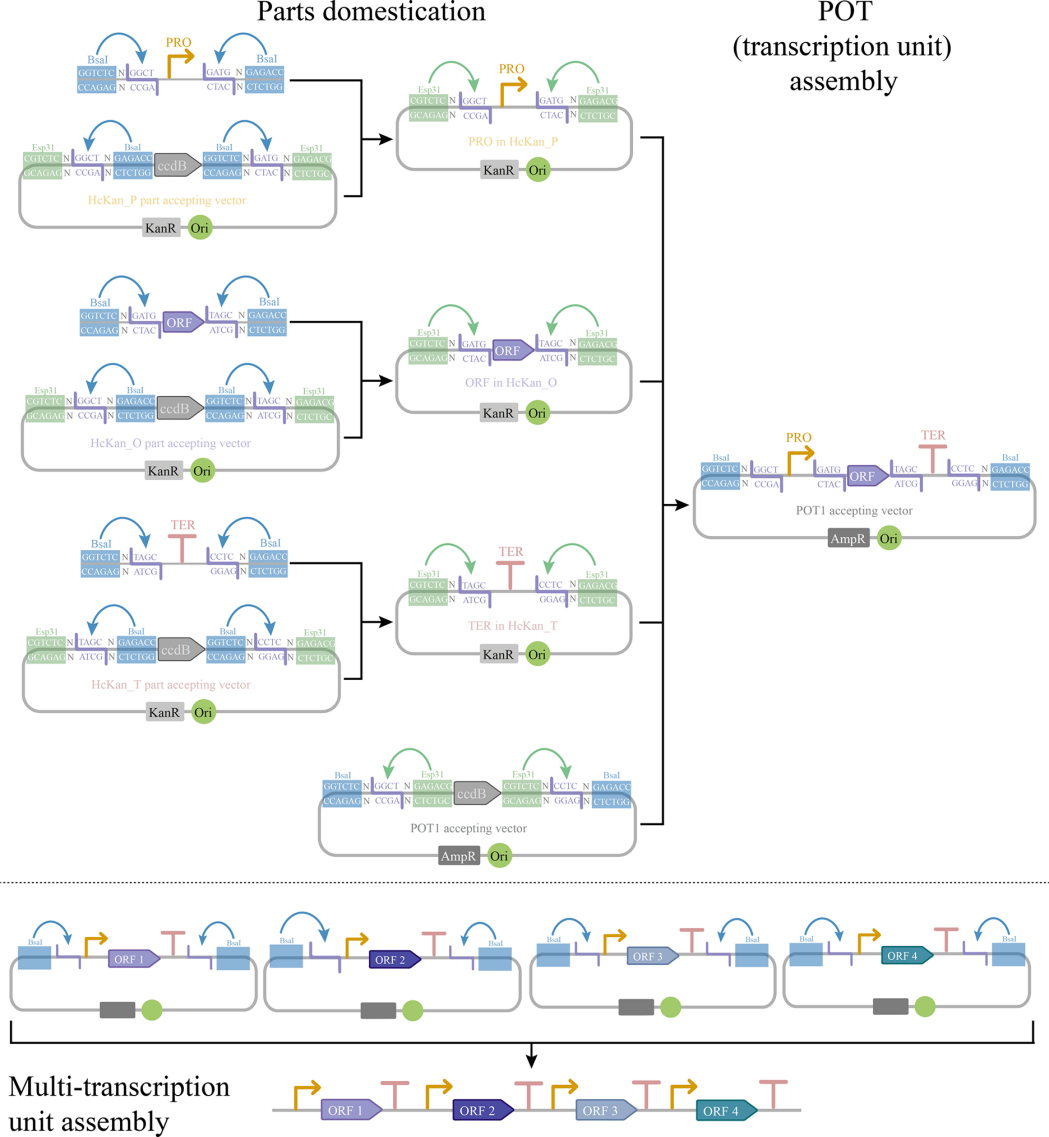
The overall scheme of multi-gene constructs *via* the YeastFab method. The functional YeastFab parts can be cloned into part accepting vectors by using the BsaI type IIS restriction enzyme. These domesticated parts can be released from part accepting vectors by using Esp3I (BsmBI) type IIS restriction enzyme and transcription units are assembled in a POT accepting vector in a promoter-ORF-terminator grammar. Following this, several transcription units in multiple POT vectors can be assembled together using BsaI.

In an attempt to overcome the limited number of parts available, Guo *et al.* (2015) developed a standardized DNA construction method called YeastFab in which hundreds of biological parts were standardized and modularized, allowing for subsequent hierarchical assembly of transcription units and ultimately multigene pathways.^65^ The method was based on the incorporation of prefixes and suffixes encoding type IIS restriction enzyme sites to parts amplified from the yeast genome. In short, defined regions in the genome (promoters (PRO), open reading frames (ORF), and terminators (TER)) were identified based on specific criteria and primers containing the required prefix and suffix were created. The desired parts could then be PCR amplified out of the yeast genome and cloned into specific “part accepting vectors”.^65^ The result was a standardized system that facilitated the expression of multicomponent exogenous pathways in *S. cerevisiae*. As such, YeastFab parts were cloned and released using the BsaI and Esp3I (BsmBI) type IIS restriction enzymes, followed by assembly *via* Golden-Gate cloning. Subsequently, multigene pathways were assembled by Golden-Gate cloning using the BsaI type IIS restriction enzyme, allowing for the integration of the pathway into a yeast genomic locus. Figure 4 demonstrates the overall scheme of the assembly of multigene constructs from biological parts. Importantly, PROs, ORFs, and TERs could be reused without the need for refactoring, thus helping researchers reconstitute and optimize multiple heterologous systems in yeast with less resources and without relying on assembly kits that require module-specific DNA designs.^65^

Using YeastFab, hundreds of yeast promoters were characterized using a yellow fluorescent protein (YFP) and mCherry expressing dual reporter plasmid.^65^ The promoters were integrated into the upstream part of YFP for expression while mCherry encoding gene was driven by TEF2 promoter as a reference. Comparing the ratios in YFP and mCherry signals, the strengths of numerous promoters were identified.^65^ Flow cytometer analysis was used to count the cells and measure the sizes so that the fluorescence ratio was calculated for each cell. Promoter activities were tested under synthetic complete medium and stress conditions such as glucose-free, nitrogen-limited, or H_2_O_2_ containing media.^65^ Moreover, the authors also constructed the β-carotene pathway using three heterologous genes (*CrtE, CrtI, CrtYB*) that were expressed by 27 different combinations of weak (P_CYC1_), medium (P_TEF2_), and strong (P_TDH3_) promoters to further evaluate the activities of promoter combinations at different strengths.^65^ The vectors produced in the study were made separately available on Addgene rather than a toolkit, which may not be ideal to improve its adoption among users. However, the code for finding the parts and designing the respective primers was made available on Github.^66^ The advantage of the strategy is that it allows a fast, one-pot assembly system. However, this system is not designed for the incorporation of an N-terminal or C-terminal tag; if required this needs to be done through using a primer or resynthesis. Another disadvantage is the user restriction to predesigned selection markers found in a minimal number of backbone vectors.

### 2.4 GoldenBraid

**Figure 5:**
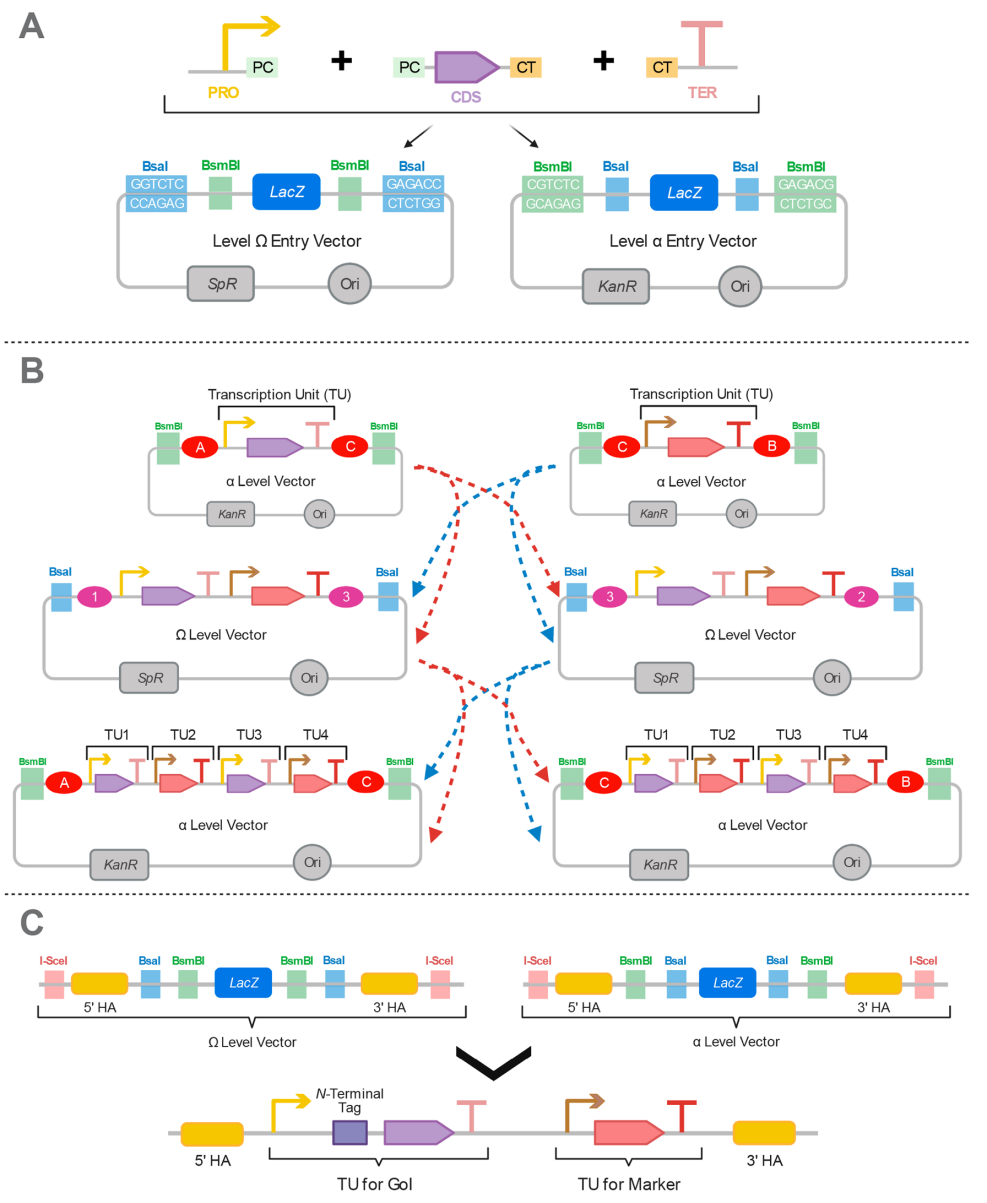
Representation of the GoldenBraid (GB) assembly method. (A) The modules, promoter (PRO), coding sequence (CDS), terminator (TER) are assembled into the level Ω (with spectinomycin-resistance gene *SpR*) or level α entry vectors (with kanamycin-resistance gene *KanR*) depending on the restriction enzyme used. The PC fragment between the PRO and CDS connects these two parts after being cut by a proper type II restriction enzyme. Similarly, the CT fragment connects the CDS and TER. Then, the transcription unit (TU) consisting of these three parts is assembled into an entry vector. *LACZ* is used as a reporter to detect the correct assemblies. (B) Two TUs can be assembled from level Ω to level α vectors or vice versa. Depending on the type II restriction enzyme used, the next level is selected. 1, 2, 3 or A, B, C in ellipse shapes represent inner cutting sites of the type II restriction enzymes so that a common sticky end can be formed with the next TU. In the first assembly, two single TUs share a common “C” sticky end, whereas “3” is shared by two double TUs in the next assembly step. More TUs can be assembled by following this order or reused as entry vectors for the next level α binary assembly. They both have a BsaI sticky end. (C) Yeast GB assembly approach used for yeast. TU contains an additional N-terminal tag for mitochondrial targeting. Also, 5′ and 3′ homology arms are added for genomic integration into the target region. The construct is linearized by the I-SceI type II restriction enzyme.

To overcome the two-part assembly limitation of BioBrick parts, the GoldenBraid (GB) type IIS restriction enzyme-based DNA assembly was developed. Originally developed for standardization of plant synthetic biology,^67^ the technique employed four destination plasmids, called pDGBs, to incorporate binarily combined multipartite assemblies including standardized DNA parts like promoters, coding sequences (CDS), and terminators (Figure 5A). Considering the position of the restriction enzymes (BsaI or BsmBI type II restriction enzymes), a double loop braid could be formed because of the binary combination of the constructs (Figure 5B). Despite hierarchical levels of MoClo assembly, a braid topology is seen between level α and level Ω of GB assembly because constructs in different levels could host each other’s parts.

This method was then adopted for *S. cerevisiae* with the Yeast GB cloning system.^68^ Yeast GB allowed integration of the constructs into two well-characterized loci, *YPRCΔ15* and *YORWΔ22*,^69^ of the yeast genome after cutting out the construct by using I-SceI (Figure 5C). With this study, a yeast toolkit was also developed which contained four integrative plasmids for each locus, nine promoters, eight mitochondrial targeting signals (MTS), one N-terminal tag, three terminators, and two dominant selective markers.^70^ Among the promoters tested in the study, P_GAL1_ was the only inducible promoter while others (P_PGK1_, P_TDH3_, P_TEF2_, P_TPI1_, P_PYK1_, P_PGI1_, P_TDH2_, P_HXT7_) were constitutive promoters with different strengths.^68^ The promoters were tested in terms of their expression activities on a heterologous gene, *nifU*, from *Azotobacter vinelandii* using two different carbon sources, glucose or glycerol.^68^ Likewise, the localization efficiencies of six native MTSs (MAM33, GLRX2, ATPA, ODPA, ODPB, and SOD2), Su9 from *Neurospora crassa*, and MTS2 from *Nicotiana plumbaginifolia* were tested in glucose or glycerol containing media. Expression and the localization of the target proteins were analyzed using SDS-PAGE and Western blotting on cytoplasmic or mitochondrial-enriched fractions of the yeast cells.^68^ The main purpose of benefiting from MTSs was ensuring proper modifications and folding of recombinant proteins expressed by Yeast GB. The GoldenBraid collection of standardized parts and tools is available online^71^ where detailed experimental tutorials are also provided.

### 2.5 CRISPR/Cas9-Based Toolkits

As CRISPR-based methods can introduce double-strand breaks that can significantly increase genome editing efficiency, it has been widely used as a marker-free tool for yeast metabolic engineering and strain development studies.^72−77^ When it comes to gene expression, genomic integration of target genes also avoids problems with variable copy numbers and instabilities associated with episomal expressions.^43,78^

### 2.6 Cas9-Based pCut Toolkit

The toolkits previously mentioned mainly resulted in a plasmid vector containing the desired construct. Alternatively, using a Cas9-based yeast toolkit has enabled the integration of genes of interest directly into the yeast genome.^79^ A total of 70 parts were developed in the initial design, including 23 Cas9-sgRNA plasmids, 37 promoters, and 10 protein tags.^80^ The 23 genomic loci, dispersed on different yeast chromosomes, were characterized in terms of integration and expression efficiency of the green fluorescent reporter protein (GFP). Also, 37 promoters with various strengths and 10 protein tags were characterized for fine-tuning of gene expression within the scope of yeast metabolic engineering studies.

**Figure 6:**
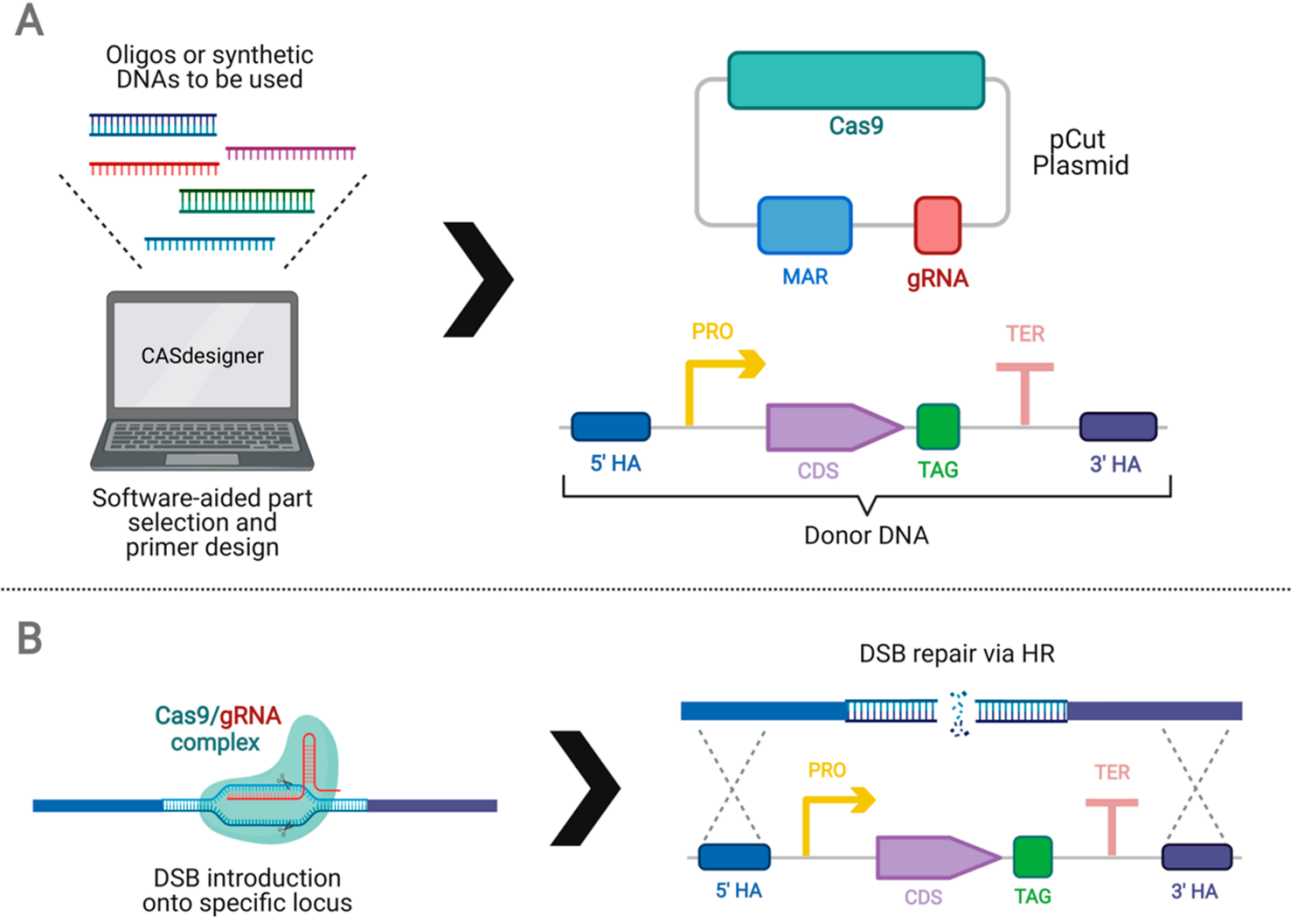
Working principle of Cas9-based pCut toolkit assembly method. (A) CASdesigner software can be used to design DNA oligos for the target construct. Donor DNA can be assembled to form a complete donor or the parts (promoter, PRO; coding sequence, CDS; terminator, TER) containing short homology fragments to adjacent fragments can be cotransformed for *in vivo* assembly. C-terminal localization signals (TAG) can be also used in donor DNA construct. (B) Cas9 and gRNA are expressed by CRISPR plasmid, and they lead to double-strand break formation on the target region of the yeast genome. Following that, donor DNA is integrated *via* homology-directed repair thanks to 5′ and 3′ homology arms.

The Cas9-based toolkit was meant to set standards for yeast-specific CRISPR applications by providing well-characterized genetic parts for integration within the genomic sites. Also, in this study, parts were designed with the provided CASdesigner tool,^81^ which assists the user in the selection of parts for their application and designs the primers needed to join the parts (Figure 6A). The donor construct can be assembled with the corresponding homology arms from the site of integration all cotransformed with a CRISPR plasmid expressing both Cas9 and gRNA. In detail, the CRISPR plasmid expression leads to a cut at the site homologous to the guide sequence, and then the donor construct is integrated at the cut site as a result of homology-directed repair (Figure 6B).

Promoter strength was also extensively characterized with a GFP reporter in three different growth media (yeast extract peptone dextrose, complete supplement medium, and yeast extract peptone galactose medium) and at different time points (4 h, 8 h, 24 h, and 48 h).^79^ Fluorescence values were determined by calculating the molecules of equivalent fluorescein (MEFL) values using a high-throughput flow cytometer platform. This allowed the researchers to have a more thorough understanding of promoter behavior. For example, they identified that while P_GAL1_ is considered one of the strongest promoters, activity level dropped in the stationary phase, after the eighth hour. This highlights the importance of dynamic promoter characterization to achieve better predictability across time periods and higher reproducibility. A modular framework was also designed for comparing protein tags to improve solubility and stability, which were tested for taxadiene production in this study and that of Nowrouzi *et al.*(82) One of the important findings given in the study was the genomic integration and gene expression efficiencies on different genomic loci as limited information is available in the literature about these for the yeast genome.^79^ The expression efficiencies of P_TEF2_ driven GFP encoding gene are given in MEFL values on 23 loci as well as the integration percentages on these locations.^79^ This information can guide the users on the selection of gRNAs or the genomic locations depending on their needs.

### 2.7 EasyClone-MarkerFree

EasyClone vectors were also adopted for CRISPR-based and marker-free genomic integration.^35^ In detail, BioBricks encoding promoters and genes of interest were generated using uracil-containing primers. The parts were then assembled into integration vectors *via* uracil-excision-based (USER) cloning.^83^ The corresponding gRNAs and Cas9 were expressed by separate plasmids, and the integration was facilitated by Cas9/gRNA complex.^84^ This strategy was implemented in a new toolkit, named EasyClone-MarkerFree Vector Set.^35,85^ This toolkit provides 11 integrative vectors similar to the original EasyClone and EasyClone2.0 vectors but with the markers removed from the vectors for marker-less integration, thereby negating the Cre-LoxP recycling step. From a Cas9 vector and 14 gRNA vectors, 11 of them are for a single genomic locus and three are for multiloci integration. Although the vectors within the EasyClone-MarkerFree Vector Set were characterized by expressing GFP on the corresponding loci, the fluorescence measurements, autofluorescence corrections, and normalizations were not clearly reported in the study. On the other hand, to test the stability of integration and expression of GFP encoding genes, five sequential passages were monitored.^35^ As a case study, a pathway to produce 3-hydroxypropionic acid was constructed integrating heterologous genes into two different haploid laboratory strains, CEN.PK113-7D, and diploid industrial strain Ethanol Red using qPCR to confirm the copy numbers of the genes integrated.^35^

### 2.8 Homologous Recombination-Based Vector Sets

Many of the toolkits discussed here, MoClo YTK, Yeast GB, Cas9-based pCut Toolkit, EasyClone-MarkerFree Vector Set, take advantage of the efficient^86^ and well-elucidated^87,88^ homologous recombination machinery in *S. cerevisiae* for genomic integration. Among the many studies using the yeast’s homologous recombination system in the last decades,^86,89^ probably the most relevant ones proving the homologous recombination capability of yeast are the assembly of the complete *Mycoplasma genitalium* genome in yeast^90,91^ and Sc2.0 project aiming to construct synthetic yeast genome.^92,93^ Gibson *et al.* showed that the assembly of large DNA fragments is more stable in *S. cerevisiae* than in *E. coli*,^90^ and they assembled 25 large DNA fragments in a single step *via* homologous recombination in yeast to produce a complete *M. genitalium* genome, 587 kb in total.^91^ The first eukaryotic synthetic genome is being constructed in Sc2.0 which is an ongoing project where the construction of seven synthetic yeast chromosomes, synII, synIII, synV, synVI, synIXR, synX, synXII, has been finished.^94^ The SCRaMbLE system employs yeast’s homologous recombination machinery to produce diverse yeast strains and synthetic genomes,^95^ and it is the main technique used as a genome minimization tool in Sc2.0.^95,96^

Relying on homologous recombination, various integrative vector sets have been developed to meet specific needs in yeast studies. To visualize different subpopulations of yeast proteins under confocal microscopy, a vector set consisting of 23 plasmids was developed containing a range of fluorescent proteins covering the visible spectrum from blue to red, epitope tags, a localization motif,^97^ and was also deposited to Addgene.^98^ Another vector set introducing C-terminal localization or purification tags *via* homologous recombination to the endogenous yeast genes was produced for biochemical, functional, or structural studies of endogenous yeast proteins.^99^ In a following study, dual tagging was used to purify functionally active large protein complexes from yeast cells.^100^ These both vector sets are available on Addgene.^101,102^

As *S. cerevisiae* has a very accurate homologous recombination system working with the “copy+paste” mechanism in the presence of a donor DNA,^86^ it has been used in many studies, especially for those involving genome design. In fact, this is such a significant feature that is one of the reasons making *S.cerevisiae* one of the main SynBio chassis.

### 2.9 Prototrophy Toolkit for Auxotrophy Compensation

For strain development and metabolic pathway construction, using auxotrophic markers is inevitable for the selection of target transformants.^103^ However, after the selection of the transformants, the plasmids containing selective markers may require removal to eliminate the burden caused by the plasmid genes.^78^ Otherwise, the cultivation of engineered auxotrophic strains would require additional supplements to be added to the culture media increasing large-scale bioprocess costs. To compensate for auxotrophy in yeast strains, Mülleder *et al.* (2016) developed a vector set, including *HIS3*, *LEU2*, *URA3*, *MET17*, or *LYS2* genes and their combinations, for *S. cerevisiae*.^104^ The kit consists of 23 single-copy centromeric plasmids that contain the selection marker genes in various combinations to compensate for their deficiencies.^105^

### 2.10 Comparison of *S. cerevisiae* Toolkits

The toolkits discussed here can be divided into two types. The first type encompasses MoClo YTK, Yeast GB, and EasyClone-MarkerFree kit which allow the construction of more complex systems such as multigene expression constructs using the characterized parts and the assembly methods mentioned. A user manual is generally provided by these toolkits explaining the design and assembly principles. A typical assembly process would take 2 to 3 days if existing parts are used or 3 to 4 days if PCR products are needed for any of these toolkits as they use similar approaches, mainly *in vitro* enzymatic treatments, for DNA assembly. The other type of toolkits provides ready-to-use plasmids for specific applications. For instance, Cas9-based pCut Toolkit provides CRISPR plasmids containing gRNAs targeting specific locations in the yeast genome. The user needs to provide their own parts to construct the repair donor DNA for genome editing. The other example is the Prototrophy Toolkit providing individual markers or their combinations in its plasmids. Therefore, an additional assembly process is not required for this type of toolkits although they are not as flexible as the other type since the latter is designed for more specific applications.

As summarized in Table 1, most of the *S. cerevisiae* toolkits reviewed here make use of the standardized Golden Gate Assembly method except for the EasyClone-MarkerFree kit that uses USER-cloning for vector assembly.^35^ Therefore, the use of the same assembly method with common Type IIS restriction enzymes, BsaI and BsmBI, can facilitate the employment of different toolkits by the same user. However, the overhangs used to insert the parts into acceptor vectors or to each other are different among the toolkits. As an example, the overhang sequences used for promoter parts in MoClo YTK, Yeast GB, and YeastFab are different from each other.^43,65,68^ Thus, the parts are incompatible between the toolkits preventing direct part exchange.

**Table 1:** Summary of *S. cerevisiae* Toolkits Comparison

	BioBricks	MoClo Yeast Toolkit (YTK)	GoldenBraid Yeast Toolkit	Cas9-based pCut Toolkit	YeastFab
number of parts	181 parts:	96 total parts:	31 total parts:	70 total parts:	>2000 total parts:
	• 20 backbone vectors	• 7 5′ assembly connectors	• 9 promoters	• 23 Cas9-sgRNA plasmids	• over 2000 promoters
	• 47 promoters	• 23 promoters	• 8 mitochondrial targeting signals	• 37 promoters	• 3 protein-coding sequences
	• 3 Kozak sequences	• 7 coding sequences	• 1 N-terminal tag	• 10 protein-localization, degradation, and solubility tags	• 2 terminators
	• 15 protein tags and motifs	• 7 terminators	• 3 terminators		
	• 92 protein-coding sequences	• 7 3′ assembly connectors	• 2 selective markers		
	• 4 transcriptional units	• 7 markers	• 8 flanking sequences for genomic integration into two loci		
	• 13 terminators	• 11 origin and homology markers			
		• 4 miscellaneous parts			
					
assembly method	Golden Gate	Golden Gate	Golden Gate	integration/homology-directed repair	Golden Gate
					
advantages	• widely used	• reusable parts	• avoids copy number variability	• avoids copy number variability	• reusable parts
	• hierarchical assembly	• fast assembly	• allows mitochondrial protein modifications	• CASdesigner tool simplifies assembly	• hierarchical assembly
		• one-pot assembly	• hierarchical assembly	• simultaneous multicopy integration	• many parts (promoter and terminator) available
		• parts compatibility			
		• hierarchical assembly			
					
disadvantages	• two parts assemblies at a time	• minimal parts available	• minimal parts available	• minimal parts available	• requires standardization of parts
	• forbidden sites				
					
multipart assembly	no	yes	yes	no	yes

In theory, an unlimited number of parts can be assembled using the GB assembly method as an infinite loop between level Ω and level α can be carried out.^68^ However, Yeast GB is specifically designed for mitochondrial protein expression with its localization signals. On the other hand, MoClo YTK provides a diverse well-characterized set of parts in all wells in the kit making it the most versatile and cost-effective toolkit among the others. Also, up to six genes can be assembled and expressed using MoClo YTK.^43^ Therefore, it has found a wide range of uses in yeast SynBio studies. Similar to MoClo YTK, the assembly method of YeastFab also allows the construction of multigene expression devices with up to six genes.^65^ However, limited types of genetic parts, individual plasmids, and validation needs are the main obstacles to its adoption.

Copy numbers of the constructs, single-cell variabilities, genomic integration rates, and a wide range of genetic parts including promoters, terminators, protein degradation tags, and origins of replication were thoroughly characterized using advanced techniques like the use of a flow cytometer in MoClo YTK.^43^ Therefore, MoClo provides useful information about many different parameters. Likewise, many promoters were characterized using a flow cytometer in YeastFab.^65^ As the Yeast GB was mainly developed to facilitate mitochondrial localization, protein expression activities of nine promoters and localization capabilities of nine mitochondrial localization signals were characterized using proteomics techniques such as SDS-PAGE and Western blotting.^68^ As expected, CRISPR-based toolkits provided genome editing efficiencies and gene expression rates on different genomic loci.^35,79^ Among them, Cas9-based pCut Toolkit also characterized 37 promoters in three different media types as well as protein tags for different purposes such as localization, solubility, and degradation.^79^ These comprehensive characterizations can give users sufficient insight into the limits or capabilities of the tools, parts, and methods provided in the corresponding studies.

The toolkits available in Addgene are provided in 96-well plates for $375 per plate as of 2022. As each well in these plates contains a single plasmid for specific parts or constructs, this is quite cost-effective compared to a single plasmid that costs $75 (2022). Among the toolkits reviewed here, MoClo YTK (96 plasmids), Yeast GB (31 plasmids), EasyClone-MarkerFree Vector Set (29 plasmids), Cas9-based pCut Toolkit (27 plasmids), and Prototrophy Toolkit (23 plasmids) are available in 96-well plate format on Addgene. Although YeastFab aimed to overcome the limited number of the parts provided in well plates, separate YeastFab plasmids are available on Addgene making this toolkit relatively costly in comparison to the previously mentioned ones. When it comes to the parts in the iGEM Registry, the iGEM headquarters no longer provide the parts themselves. Instead, the registered teams or laboratories are given some free quotas to be used for the synthesis of the fragments of interest by the iGEM sponsors. This makes the use of these parts more limited for general use by the SynBio community.

Overall, each yeast-specific toolkit has been developed to meet particular demands such as mitochondrial targeting,^68^ auxotrophy compensation,^104^ or genome editing,^79^ as mentioned above. Among the toolkits, MoClo YTK is a widely adopted one addressing various needs from constructing complex expression systems to genomic integration features with its relatively wide range of well-characterized genetic parts. Although the iGEM repository and YeastFab provide more parts than the others, their parts require further characterization and validation for being adopted by the yeast SynBio community. Perhaps, one of the main limitations of the characterization studies is the use of limited conditions and limited yeast strains. Nevertheless, many independent studies employing these toolkits for different strains and in different conditions have been reported.^59,63^

## 3 Toolkits for Nonconventional Yeast Species

In addition to the toolkits/standardized parts developed for *S. cerevisiae*, toolkits for standardization have also been developed for other yeast species, extending the scope of possible applications for yeast synthetic biology:

- The methylotrophic *Komagataella phaffii (Pichia pastoris)* has been widely used as an alternative chassis for SynBio as it can convert multiple substrates into various value-added compounds, while also achieving high cell densities with high growth rates on inexpensive media.^106−109^ Added to these benefits, the modularization of secretion machinery can make this organism more efficient for producing and secreting desired industrial products.^110^ Accordingly, SynBio tools have been developed and genetic parts have been characterized to engineer *K. phaffii*.^108^
- *Yarrowia lipolytica* is an oleaginous yeast species that is also widely used for metabolic engineering and biotechnological production especially for lipid-based, high-value products thanks to its lipid storage capabilities.^111−114^ It has been also studied for the production of nonlipid chemicals such as sugar products, organic acids, and aromatic compounds.^115−118^ For this reason, the availability of genetic tools plays an important role in the development of SynBio feasibilities for this important host.^119^
- *Kluyveromyces marxianus* is another nonconventional yeast with favorable characteristics, like utilizing a wide range of sugars,^120,121^ hydrolyzing complex plant fructans, and growing at relatively high temperatures (>40 °C).^122,123^*K. marxianus* can also be used as an alternative platform for the production of valuable compounds such as biofuels, fragrances, and flavors.^124,125^ Therefore, characterized parts and tools are needed to accelerate metabolic engineering in *K. marxianus*.

### 3.1 *Komagataella phaffii* SynBio Toolkits

#### 3.1.1 Moclo *Pichia* Toolkit

**Figure 7:**
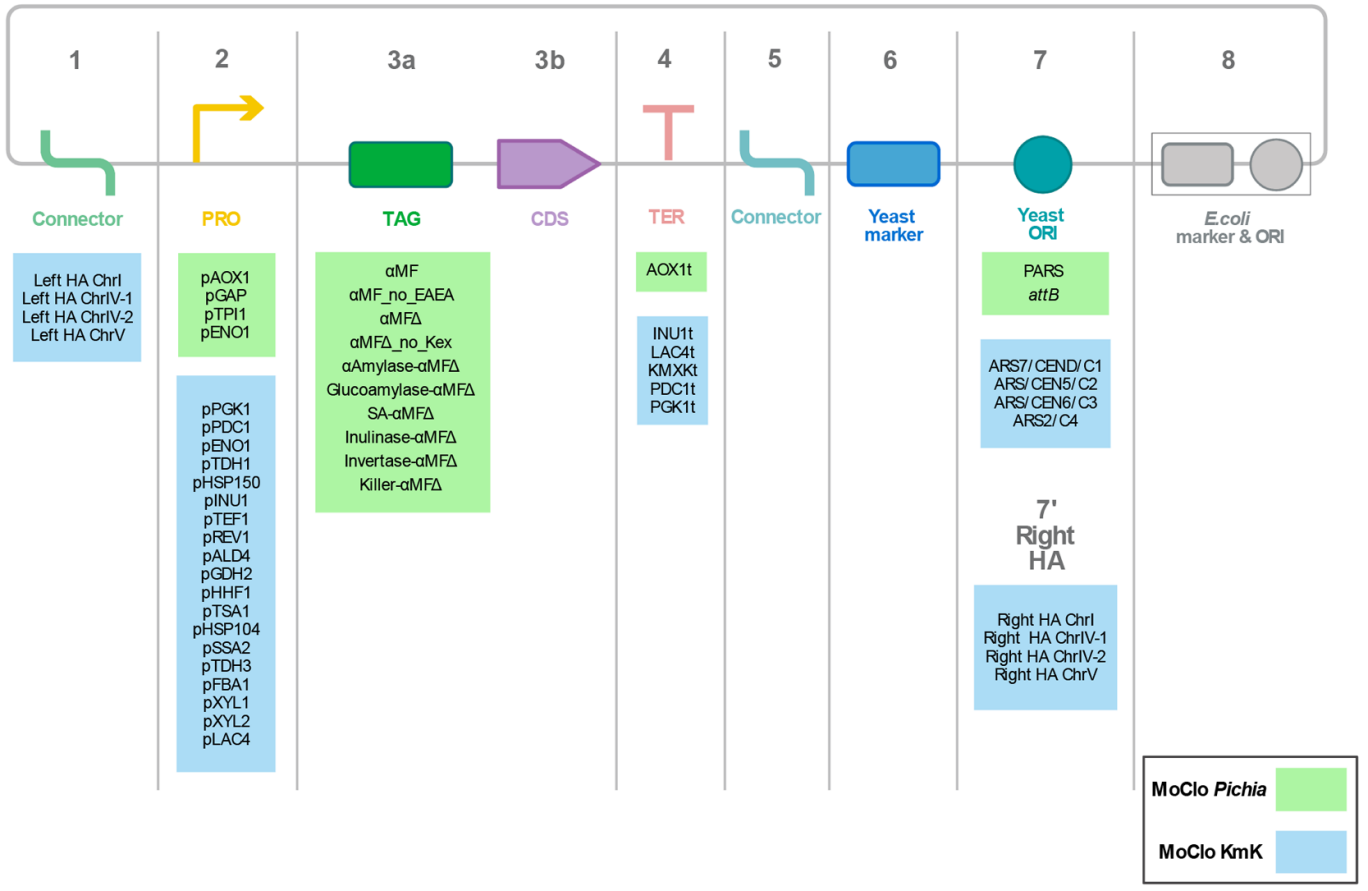
Toolkits adapted from MoClo YTK for *K. phaffii* and *K. marxianus*. Additional parts (shown on green background) including secretion signals (TAG), promoters (PRO), and terminators (TER) have been characterized for *K. phaffii* and the parts are provided with the MoClo *Pichia* toolkit. Additionally, *K. marxianus*-specific parts (shown on blue background) containing 19 promoters and 5 terminators have been characterized. Alternatives of yeast origin of replications (ORI) have also been added to the kit (*K. marxianus* kit, KmK) along with four homology arms for genomic integration of the constructs (HA, homology arm; Chr, chromosome; MF, mating factor).

To optimize the protein secretion *viaK. phaffii*, Obst *et al.* (2017) developed a *K. phaffii*–specific toolkit (MoClo *Pichia*)^126^ which is also compatible with the hierarchical MoClo method (Figure 7).^127^ To evaluate the performance of the parts, the secretion and expression efficiencies of 124 constructs were characterized using red fluorescence protein (RFP) and GFP reporters. Constructs were also compared for efficiency using integration-based or plasmid-based delivery methods.^127^

This study emphasized the importance of a diverse library for creating optimal secretion constructs as there was a high level of variability,^127^ and it could be unclear how parts affected one another. The new elements of the library consisted of 17 control elements, 4 promoters, 10 secretion tags, 1 terminator, and 2 origins of replication (Figure 7). Results showed that the secretion efficiency was independent of the downstream coding sequence and that the secretion constructs could be made in weeks rather than months with this standardized method.

#### 3.1.2 Golden*Pi*CS Toolkit

**Figure 8:**
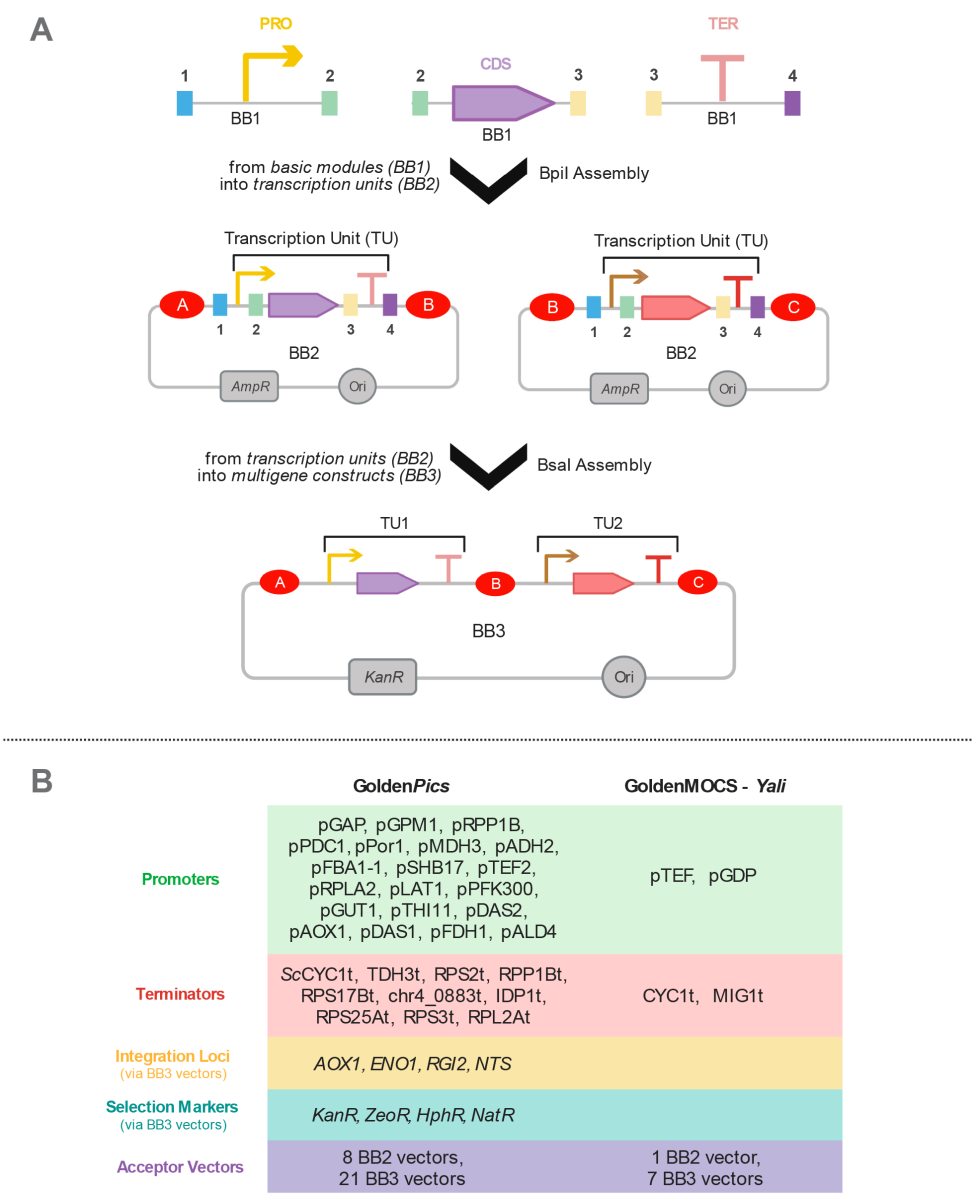
GoldenMOCS assembly system and the relevant toolkits. (A) Similar to MoClo, the basic modules (promoter, PRO; coding sequence, CDS; terminator, TER) are kept in BB1 vectors and they are assembled using BpiI type II restriction enzyme to create complete transcription units (TU) in BB2 vectors. Multigene constructs can be created *via* BsaI type II restriction enzyme into BB3 vectors. (B) Species-specific parts have been characterized for *K. phaffii* (Golden*Pics*) and *Y. lipolytica* (GoldenMOCS-*Yali*) using the GoldenMOCS assembly approach.

Sarkari *et al.* (2017) developed a flexible DNA assembling method called GoldenMOCS (Golden Gate derived Multiple Organism Cloning System), derived from the Golden Gate-based MoClo hierarchical DNA construction approach.^128^ GoldenMOCS consists of three levels of DNA construction. The basic parts consisting of promoters, CDSs, and terminators are categorized in level 1. Assembly of level 1 parts leads to the construction of a single expression cassette in level 2, and level 3 is obtained through assembling multiple expression cassettes (up to eight cassettes) like a whole metabolic pathway (Figure 8A). While promoters and terminators are species-specific, the core parts, CDSs, can be used from various microbial species, making GoldenMOCS a more versatile approach.

Later, Prielhofer *et al.* (2017) developed another toolkit for *K. phaffii* called Golden*Pi*CS, based on GoldenMOCS.^129^ This toolkit^130^ contains 20 promoters, 10 transcription factors, 4 genome integration loci, and 4 resistance marker cassettes (Figure 8B). Similar to GoldenMOCS which enables the expression of eight genes simultaneously, Golden*Pi*CS can be also used for metabolic pathway construction and recombinant protein production in *K. phaffii*.

The parts in the Golden*Pi*CS toolkit were initially characterized with enhanced GPF (eGFP) under varying conditions, with excess glucose and glycerol present, limited glucose, and methanol feed.^129^ By adding such conditions as variables for characterization, a more thorough understanding of the behavior of the parts is generated.

#### 3.1.3 Yeast Secrete and Detect

Recently, a modular protein secretion toolkit has been developed for two yeast species, *K. phaffii* and *S. cerevisiae*, using Golden Gate assembly.^131^ Originally, the study aimed to develop a secretion system for fungal unspecific peroxygenases (UPO, EC 1.11.2.1) catalyzing hydroxylation, epoxidation, aromatization, sulfoxidation, N-oxygenation, dechlorination, etc. reactions.^132^ For the extracellular expression of UPOs, three modules were used. The first module is the N-terminal signal peptide, which is responsible for the secretion and contains a start codon. The second module is a UPO gene that does not include the start or stop codon. The third one is the C-terminal tag for purification and GFP reporter.^131^ Researchers achieved 24 mg/L secreted UPO enzyme through *K. phaffii* using this design. The toolkit developed in this study has a collection of 42 plasmids available on Addgene,^133^ including 17 signal peptides, 8 protein-coding genes expressing UPOs, and 7 C-terminal protein tags.

### 3.2 *Yarrowia lipolytica* SynBio Toolkits

#### 3.2.1 GoldenMOCS-*Yali*

GoldenMOCS was also used to create a vector set for *Y. lipolytica*.^134^ The vectors included extrachromosomal vectors allowing expression of up to four transcriptional units that could also be used in conjunction with CRISPR/Cas-9 to integrate the sequences into the genome. With these vectors, *Y. lipolytica*-specific parts including two replication sequences, two promoters, and two terminators were also added to the GoldenMOCS part depository^135^ (Figure 8B). As case studies, Egermeier *et al.* (2019) overexpressed glycerol kinase (*GUT1*) and deleted the *LEU2* gene using GoldenMOCS-*Yali*, resulting in enhanced erythritol and citric acid production from glycerol.

#### 3.2.2 EasyCloneYALI

Quite similar to *S. cerevisiae*, EasyCloneYALI, based on EasyClone vectors, was also developed for *Y. lipolytica*. Two EasyClone vector sets,^136^ one for marker-dependent integrative vectors and the other for CRISPR-based marker-free integration, were created using standardized BioBrick parts while USER cloning was used for the assembly.^137^ Integrative vectors were constructed in a way that they contained a selection marker (*URA3* auxotrophic marker, nourseothricin, or hygromycin resistance marker) with loxP sites for removal of the markers using Cre recombinase.^138^ Akin to the original EasyClone vectors, the EasyCloneYALI vector sets targeted 11 characterized genomic regions.^137^ The genomic loci were characterized by integrating GFP into each locus. In the CRISPR-based vector set, Cas9 expression and gRNA expression vectors were also provided. The integration efficiency of the CRISPR system was tested on 11 loci using marker-free linearized integrative vectors. Although more than 80% integration was detected on 5 out of 11 loci, the integration rate was not high for the other loci.^137^

#### 3.2.3 *Y. lipolytica* Golden Gate Toolkit

In another study, Larroude *et al.* (2019) developed a Golden Gate-based *Y. lipolytica* toolkit which included 64 Golden Gate bricks.^139^ Using these bricks, nine promoters (six constitutive and three inducible) with different strengths, five different terminators, three auxotrophic markers, two antibiotic resistance markers, and one metabolic marker (invertase from *S. cerevisiae*) were tested and characterized with three fluorescent reporter proteins. The toolkit^140^ also allows genomic integration by its flanking regions targeting zeta sequences that are found at the terminals of repetitive retrotransposons, Ylt1, in the *Y. lipolytica* genome.^141^ As a proof of concept, the xylose utilization pathway consisting of three genes, xylitol dehydrogenase, xylose reductase, and xylulokinase, was constructed to demonstrate the utility of the toolkit. It was detected that 79% of the transformants grew on the media containing xylose as its sole carbon source.^139^

#### 3.2.4 *Y. lipolytica* Cell Atlas

To observe the localization of biosynthetic enzymes and dynamics of endogenous organelles in live *Y. lipolytica* cells, Bredeweg *et al.* (2017) developed a suite of isogenic strains named *Y. lipolytica* Cell Atlas.^142^ Researchers first constructed nonhomologous end-joining (NHEJ)-deficient auxotrophic strains to increase targeted integration yield through homologous recombination.^143^ Following this, GFP-tagged enzymes involving in triglyceride biosynthesis were episomally expressed to monitor their cellular localizations.^142^ The organelle-specific genes were also tagged with GFP at their endogenous genomic loci to define the organelle dynamics in the cells.^142^ The strains constructed for each particular organelle, nucleus, mitochondrion, peroxisome, lipid droplet, endoplasmic reticulum, vacuole, and Golgi apparatus, are available at the Fungal Genetics Stock Center.^144^

### 3.3 *Kluyveromyces marxianus* SynBio Toolkit

Based on the MoClo YTK plasmid construction standard, the *Kluyveromyces marxianus* Kit (KmK) was developed which contains over 30 characterized parts including strong, medium, weak, and inducible promoters, five different terminators, and four different replication origins.^145^ Promoters and terminators were initially characterized by expressing YFP reporter on centromeric plasmids to eliminate copy number bias. Genome editing efficiency of a single Cas9/gRNA coexpression plasmid was also tested on the *LAC4* gene due to its relatively easy screening method where the mutants did not convert X-gal to blue dye.^145^ The plasmids used in the study are also available on Addgene.^146^

## 4 Utilization of Yeast SynBio Toolkits for Automated and High-Throughput Studies

Modular assembly of standardized genetic parts provides flexibility in the design of expression constructs and facilitates studies to characterize new coding and regulatory sequences allowing us to equip cells with new or improved functionalities. However, manual approaches to design and domesticate sequences and pipet different combinations of parts are a laborious, repetitive, error-prone, and time-consuming process.^147^ Therefore, manual protocols are particularly unsuitable for generating large-scale combinatorial libraries or generating and testing randomized designs in iterative design-test-build cycles. Although most of the yeast toolkits operate on similar principles, the suitability of individual toolkits for automation can be further characterized using a DNA assembly metric (Q-metric), which assesses the cost and time benefit of automated vs manual assembly protocols.^147^

Modular assembly methods that utilize one-pot digestion-ligation reactions are well suited for automation and miniaturization using microfluidics, liquid handling robots, acoustic droplet ejection (ADE) dispensers, and automated colony pickers.^148,149^ Automated pipelines for DNA assembly have already been described for several methods,^149^ including the yeast YTK toolkit^40^ and similar to modular assembly toolkits for plant and mammalian toolkits.^150,151^ Furthermore, miniaturized protocols that use ADE dispensers to set up submicroliter reaction mixes are well established and further reduce costs and resource usage.^152^ This proved particularly useful during the SARS-CoV-2 pandemic as there were, and still are, severe delays in the supply chains of common laboratory plastic ware.

**Figure 9:**
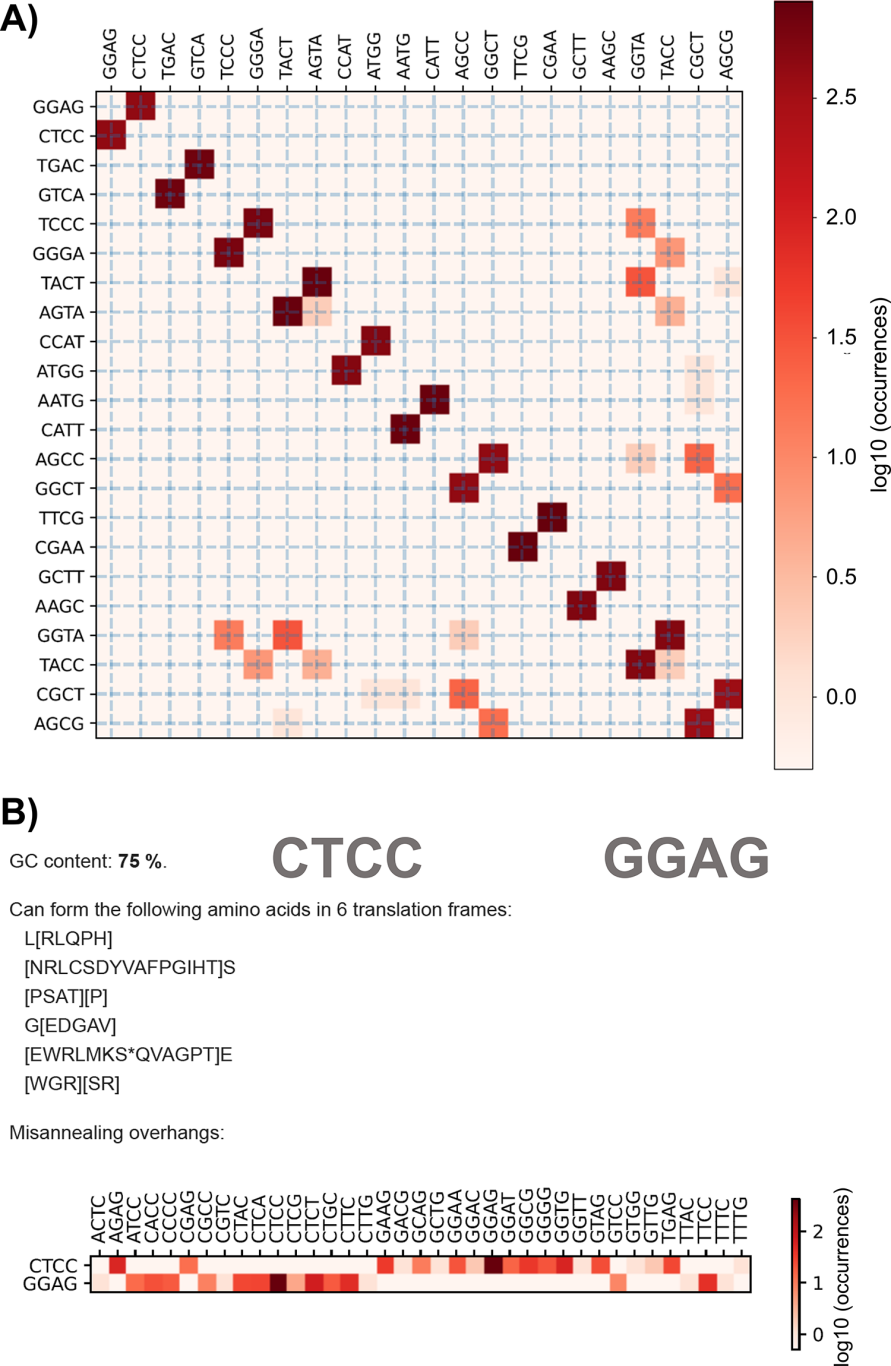
Sample overhang misannealing analysis of Tatapov used for yeast GB toolkit. (A) General evaluation of overhang misannealings in the target set. The squares outside the diagonal square pairs show the cross-talking, so misannealing risks. In addition, lighter square pairs on the diagonal show weak self-annealings, so the risk of having no assembly. (B) Detailed evaluation for the CTCC overhang.

Biofoundries have played a key role in providing researchers unprecedented access to equipment and automation infrastructure. The high-throughput DNA assembly process is streamlined through highly automated platforms like the one found at the Edinburgh Genome Foundry (Figure 9). The modularity of the process facilitates the reuse (and exchange) of parts within the research community.^153^ However, the uptake of low-cost automated liquid handling solutions would further support the use of automation technology for laboratories with limited funding.^154,155^ End-to-end automated protocols have already been developed on Opentrons OT-2 systems for BASIC and MoClo assembly methods that could be adapted for other toolkits with relative ease.^154,156^

Complementing automation, biofoundries also develop software packages that assist in all stages of the design and build. They enable validating and preparing batches of partial sequence files for BioBricks, MoClo, and other toolkits.^157,158^ Cloning simulation software generates sequence files for the resulting plasmids, allowing prevalidation of the assemblies. These packages are usually released under a free and open-source license, which enables incorporation into more comprehensive tools, such as Web sites that provide graphical user interfaces for these packages for noncomputational users (https://cuba.genomefoundry.org/) or systems that model DNA supply and assembly networks to create assembly strategies (https://dnaweaver.genomefoundry.org/). Biofoundries also provide various tools for generating robot instruction files for performing assemblies^159^ and then validating them by Sanger sequencing^160^ or single-molecule sequencing.^161^ Finally, a critical factor in the success of an assembly is the selection of overhangs. The NEB Ligase Fidelity tools (https://ligasefidelity.neb.com/)^162^ and Tatapov and Overhang packages can be used to evaluate the kits. These utilize experimental overhang misannealing data to select the best set of parts/overhangs, as given as an example in Figure 9. The Kappagate package (https://edinburgh-genome-foundry.github.io/) predicts the percentage of good clones using the misannealing data.

## 5 Conclusion

This manuscript presents a review of studies which aim to develop toolkits and DNA assembly methods for yeast, concluding their foundational contribution to the definition of a collection of standards for SynBio in yeast. Yeasts are a very important family of industrial production hosts and have compelled their establishment as an indispensable chassis in SynBio.

The BioBrick yeast library, being fully community-driven and extensive, lacks characterization data for the behavior of the parts. In contrast, the high quality and depth of information available for each part can be enjoyed in newly developed toolkits like MoClo YTK and GoldenMOCS. As a result, these easy-to-use and characterized kits are widely adopted by the SynBio community and are accessible through Addgene and similar repositories.

A challenge for all standards is maintaining and updating the toolkits as the field advances. For example, while some widely used toolkits like MoClo and GoldenMOCS have been adapted to multiple species, they lack version updates. MoClo YTK provides clear and detailed user guide documentation, but online platforms presenting up-to-date parts or methods are not available for these toolkits. At this point in time, BioBricks in the iGEM catalog benefits from regular updates and, for now, the best example of version updates for characterized parts is the GoldenBraid online system providing updated information about part collections and methods. Currently, GoldenBraid version 4.0 is available on this platform with detailed tutorials and updates for users. Confirmation of a standard’s worth comes in its adoption by practitioners and, as such, supporting information is very valuable in user recruitment and retention.

There has been a trend to develop more novel toolkits, aiming specifically at genome engineering applications using CRISPR-based systems, such as the Cas9 pCut toolkit. Newer standardization parameters such as loci characterization and DNA integration efficiency have been added to the toolkits, allowing for rapid genome engineering applications while leveraging recent development in complementary technologies such as automation and emerging biofoundries. Robotic platforms and open-source software can also be adapted for automated protocols as well as quality control software for high-throughput studies involving the compatible yeast SynBio toolkits. New emerging yeast chassis such as *Komagataella phaffii*, *Yarrowia lipolytica*, and *Kluyveromyces marxianus* have greatly benefited from the toolkits developed for *Saccharomyces cerevisiae*, which will allow these yeast chassis to be more widely adopted by practitioners across academia and industry. These early examples of SynBio standards will contribute to the acceleration of translation of yeast SynBio into sustainable, real-world products and applications in the next decade.

## Abbreviations

SynBio: synthetic biology
PRO: promoter
RBS: ribosomal binding sequence
CDS: coding sequence
ORF: open reading frame
TER: terminator
TU: transcriptional unit
ORI: origin of replication
GFP: green fluorescence reporter protein
USER: uracil-excision-based
iGEM: International Genetically Engineered Machine
MoClo: modular cloning systems
GB: GoldenBraid
YTK: yeast toolkit
GoldenMOCS: Golden Gate derived Multiple Organism Cloning System
